# Morphological and molecular assessment of muscle metacercariae infecting tench *Tinca tinca* from fish farms and wild populations in Germany

**DOI:** 10.1038/s41598-025-09396-y

**Published:** 2025-07-03

**Authors:** Claudina Mata-Marcano, Matthias Stöck, Klaus Knopf

**Affiliations:** 1 Department of Fish Biology, Fisheries and Aquaculture, Leibniz Institute of Freshwater Ecology and Inland Fisheries, Müggelseedamm 310, 12587 Berlin, Germany; 2 Albrecht Daniel Thaer Institute for Agricultural and Horticultural Sciences, Faculty of Life Sciences, Humboldt University Berlin, 10115 Berlin, Germany; 3 Present Address: Laboratorio de Parasitología y Medicina de la Conservación, Escuela Nacional de Estudios Superiores Unidad Mérida, Universidad Nacional Autónoma de México (UNAM), Mérida, Mexico

**Keywords:** Metacercariae; fish-borne zoonotic trematodes; digenea, *Pseudamphistomum truncatum*, *Paracoenogonimus ovatus*, *Hysteromorpha triloba*, Biodiversity, Parasitology

## Abstract

Fish can be infected with metacercariae (the final larval stage) of different species of potentially zoonotic digenetic trematodes (flukes). The fish-borne zoonotic trematodes thus compromise food safety and present a major threat for human health. Reducing the risk of human infections requires careful assessment and accurate taxonomic identification of these parasites. Here, we analysed metacercariae in muscle tissue of tench (*Tinca tinca*), sampled between March and September 2022 from three fish farms and three natural waterbodies in Germany. Whenever possible, we combined morphological and molecular data from the very same individual metacercariae using mitochondrial cytochrome oxidase *c* subunit 1 (cox1) and nuclear internal transcribed spacer 1 (ITS1) for species identification. Three morphotypes of metacercariae were found in the muscle of tench, corresponding to the trematode species *Pseudamphistomum truncatum* Rudolphi, 1819 (Opisthorchiidae), *Hysteromorpha triloba* Rudolphi, 1819 (Diplostomidae), and *Paracoenogonimus ovatus* Katsurada, 1914 (Cyathocotylidae). The high prevalence of metacercariae of *P. truncatum* and *P. ovatus*, both with zoonotic potential, poses a risk for human infections if undercooked or raw tench is consumed.

**Supplementary Information:**

The online version contains supplementary material available at 10.1038/s41598-025-09396-y.

## Introduction

Fish can serve as the second intermediate host of the metacercariae of many digenetic trematode species. Fish-borne trematodes that parasitize fish as their second intermediate host comprise about 12 systematic families^1^. Among them, the species with zoonotic potential belong to seven families: Opisthorchiidae, Clinostomidae, Heterophyidae, Echinochasmidae, Isoparorchiidae, Troglotrematidae and Plagiorchiidae^2^. Food-borne trematodiases are considered neglected parasitic diseases^3^ even though 750 million people are at risk^4^. The risk of human infections is related to social and cultural traits associated with certain dietary habits as the predilection for raw or undercooked fish^5^. The opisthorchiid liver flukes *Opisthorchis felineus* Rivolta, 1884, *Opisthorchis viverrini* Poirier, 1886 and *Clonorchis sinensis* Cobbold, 1875 are considered the most concerning fish-borne zoonotic trematodes^6^. Opisthorchiasis is a public health problem particularly in Asia, but also in Russia and the Ukraine^7^, whereas recent reports from other European countries are rare^8^.

Reliable identification tools are needed to assess the distribution of trematode infections of fish, particularly due to their zoonotic potential and consequent relevance to food safety, economy, and public health^9,10^. The identification to the species-level of most digeneans is based almost exclusively on morphological features of adult animals^11^. Only few morphological descriptions of immature stages are available and the poor development of certain organs in larval stages complicates their identification^10^. Moreover, intraspecific morphological variation of trematodes and accumulating evidence for widespread occurrence of cryptic species in this group increases the chance for errors^12,13^. Therefore, pure morphological identification of metacercariae should be taken with caution and ideally complemented with molecular techniques or the examination of adult specimens directly related to the metacercariae^14^. However, so far, molecular approaches are not exempt from challenges including the availability and taxonomic assignment of sequences in databases, and the difficult delimitation of the genetic variation among taxa and across taxonomic levels, depending on the genetic markers and the taxa^15^. Consequently, combination of morphological and molecular data, along with additional sources of information, such as biogeography, life history traits and ecology under one conceptual framework, thus termed integrative taxonomy^16^, has been recognized as a promising approach for the delimitation of trematode species^13,17,18^.

In Berlin and Brandenburg (Germany), metacercariae of *O. felineus* have been found in common roach (*Rutilus rutilus* Linnaeus, 1758), rudd (*Scardinius erythrophthalmus* Linnaeus, 1758), common bleak (*Alburnus alburnus* Linnaeus, 1758), common bream (*Abramis brama* Linnaeus, 1758), white bream (*Blicca bjoerkna* Linnaeus, 1758) and blue bream (*Ballerus ballerus* Linnaeus, 1758); whereas the adult trematodes have been reported in red foxes (*Vulpes vulpes* Linnaeus, 1758), muskrats (*Ondatra zibethicus* Linnaeus, 1766), and cats (*Felis catus* Linnaeus, 1758)^19–24^.

Remarkably, these studies do not include tench, although it is an important secondary species in pond culture^25^ and, while not among the top targeted species, highly valued by anglers^26^. There are only few systematic studies on the parasitofauna of tench in Germany, with only two trematode species, *Tylodelphys clavata* and *Asymphlodora tincae*, and 18 species of other metazoan parasites previously reported [27 and references therein]. None of these studies have so far looked specifically for potentially zoonotic metacercariae in the muscle of the tench. Due to two outbreaks of human infections with *O. felineus* reported after the consumption of marinated tench (*Tinca tinca* Linnaeus, 1758) from a lake in Italy^27,28^, and the knowledge gap about the parasitofauna of tench in Germany^29^, the aim of this study was to determine the diversity of trematode species in the muscle of tench and the occurrence of fish-borne zoonotic trematodes, using integrative morphological and molecular analyses of the same individual metacercaria in tench collected from fish farms and natural waterbodies of Germany.

## Results

### Fish samples and parasitological parameters

The mass of tench differed significantly between the localities sampled during this study (Table 1; Kruskal-Wallis test: H (_5_) = 61.187, *p* < 0.001).

**Table 1:** Sample size and mass (g) of the tench examined, prevalence (%) and mean intensity of opisthorchiid metacercariae, and prevalence of diplostomid and cyathocotylid metacercariae. The 95% confidence interval is shown in parentheses, different superscripts indicate significant differences (Kruskal-Wallis and Dunn’s posthoc test for fish mass and mean intensity, Chi-square tests with Bonferroni correction for prevalence; *p* < 0.05).

Locality	Fish farm 1 (Brandenburg)	Fish farm 2 (Saxony)	Fish farm 3 (Bavaria)	Lake Müggelsee	Lake Blankensee	River Spree
No. of samples	10	22	11	22	22	10
Fish mass mean ± SD	439 ± 76 ^a^	88 ± 77 ^b^	509 ± 148 ^a^	431 ± 351 ^ac^	179 ± 105 ^b^	156 ± 94 ^bc^
Opisthorchiidae						
Prevalence	20 (6–51) ^ab^	0 (0–15) ^a^	0 (0–26) ^a^	50 (31–69) ^b^	45 (27–65) ^b^	100 (72–100) ^c^
Mean intensity ± SD	1.0 ± 0.0 (1)	-	-	3.5 ± 2.7 (1–9)	2.0 ± 2.2 (1–8)	5.9 ± 4.8 (1–15)
Diplostomidae						
Prevalence	40 (17–69)	14 (5–33)	9 (2–38)	45 (27–65)	18 (7–39)	0 (0–28)
Cyathocotylidae						
Prevalence	100 (72–100) ^a^	86 (67–95) ^a^	0 (0–26) ^b^	59 (39–77) ^ac^	36 (20–57) ^bc^	100 (72–100) ^a^

**Fig. 1:**
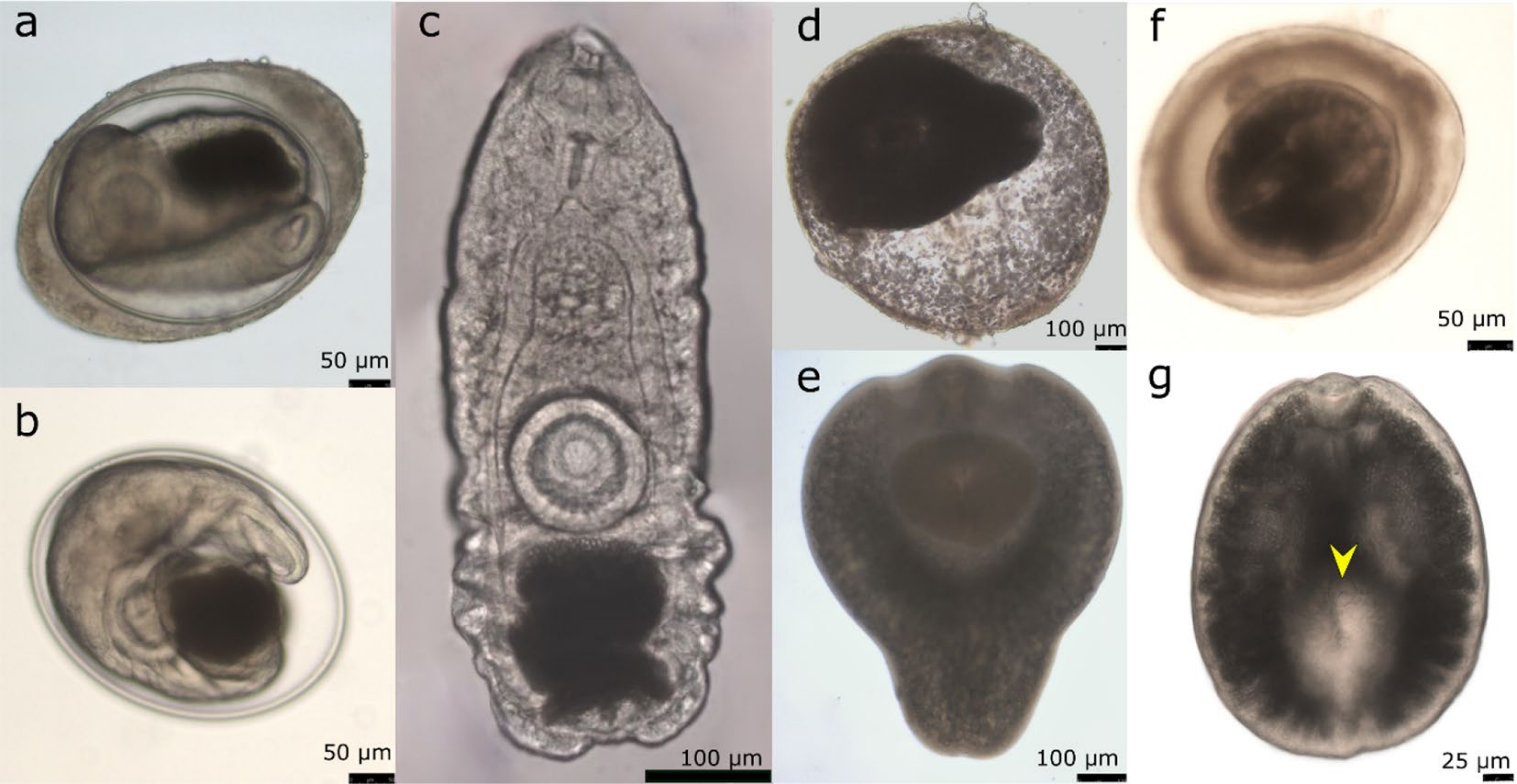
Microphotographs of live (**a**,** b**,** c**) opisthorchiid, (**d**,** e**) diplostomid and (**f**,** g**) cyathocotylid metacercariae isolated from tench. (**a**,** b**,** d**,** f**) cysts, (**b**) cyst with outer, host-derived layer removed, (**c**,** e**,** g**) excysted metacercaria. Arrowhead: ventral sucker.

Three different types of metacercariae were found in the musculature of tench (Fig. 1). Considering all the sampling sites, metacercariae were detected in the muscles of 77 tench (79.4%). From the infected fish, 42 tench (54.5%) harbored only one type of metacercaria. Co-infections with two types of metacercaria were detected in 32 tench (41.6%), and three tench (3.9%) were infected with all three species of metacercaria detected during the study. Based on the morphology of cysts and excysted metacercariae, these were assigned to the families Opisthorchiidae, Diplostomidae and Cyathocotylidae.

Opisthorchiid metacercariae were found in fish from all natural waterbodies, but only in one fish farm, with prevalences ranging from 20 to 100%, and overall, more prevalent in natural waters (Table 1). Diplostomid metacercariae were likewise detected in tench from all sampling sites, excluding River Spree, with prevalences ranging from 9 to 48%. Cyathocotylid metacercariae were only absent in tench from fish farm 3, whereby the prevalences showed no pattern in terms of occurrence in fish farms or natural waters and ranged between 36% and 100%.

The mean infection intensity of opisthorchiid metacercariae ranged from 1.0 in fish farm 1 to 5.9 in River Spree (Table 1) and although highly variable, no significant differences between localities were detected (Kruskal-Wallis H-test: H _(3)_ = 7.767, *p* = 0.051).

### Morphological description of opisthorchiid metacercariae

The cysts were round and composed of two distinct layers. The outer layer was light yellow-brownish in color, and the inner layer was hyaline (Fig. 1a, b). Within the cyst, the metacercaria was usually folded and its characteristic dark excretory bladder was easily observed. The body was flat, elongated, slightly oval and tapering anteriorly (Fig. 1c). The integument was covered with small spines. The subterminal oral sucker was slightly smaller than the ventral sucker. The pharynx was followed by a short oesophagus and the intestinal caeca that reached up to the posterior end of the excretory bladder. Below the bifurcation of the intestines the cephalic glands were visible. The ventral sucker was located toward the upper end of the posterior half of the body. The excretory bladder placed below the ventral sucker, occupied almost one third of the total body length and was filled with small, rounded granules. Morphometric data of metacercariae are given in Table 2.

**Table 2:** Morphometric data (in µm) of metacercariae from the present study.

	Opisthorchiid metacercariae		Diplostomid metacercariae		Cyathocothylid metacercariae	
	n	Mean (range)	n	Mean (range)	n	Mean (range)
Cyst ^1^ (length x width)	56	385 (243–456) x 290 (182–397)	1	1183 × 1087	21	312 (239–375) x 291 (231–325)
Body (length x width)	56	629 (374–1011) x 187 (101–266)	23	906 (601–1360) x 651 (485–828)	28	322 (232–469) x 240 (173–343)
Oral sucker	56	89 (52–110)	23	74 (48–104)	28	45 (36–63)
Ventral sucker	56	99 (55–120)	20	88 (55–116)	13	24 (18–30)
Pharynx	36	29 (17–45) x 26 (13–35)	-	-	8	30 (21–40) x 25 (15–35)
Brandes organ	-	-	22	223 (141–317) x 193 (145–246)	20	97 (78–120) x 82 (62–102)

^1^ Measures of the inner layer of the cyst wall.

### Molecular characterization of opisthorchiid metacercariae

Thirty newly generated partial sequences of the mitochondrial cox1 gene were aligned with sequences of the cox1 gene used for phylogenetic study of the family Opisthorchiidae by Sokolov et al.^30^. The analysis involved 84 sequences (Supplementary table S1), and the alignment was 1010 bp long.

Sequences of *Pseudamphistomum truncatum* (KP869078 and KP869080 to KP869085) were the most closely related retrieved by BLAST searches in NCBI (GenBank, consulted on 15/01/2024), when sorted by the percentage of identity with similarity values between 100% and 98.3%, although the query coverage was only 30%. Due to this low alignment coverage with *P. truncatum* sequences, BLAST search results were sorted by e-value and the best matches corresponded to *Metorchis xanthosomus* (NC079699) and *M. bilis* (NC079698), with similarity values between 86 and 87%, and 84–85%, respectively.

**Fig. 2:**
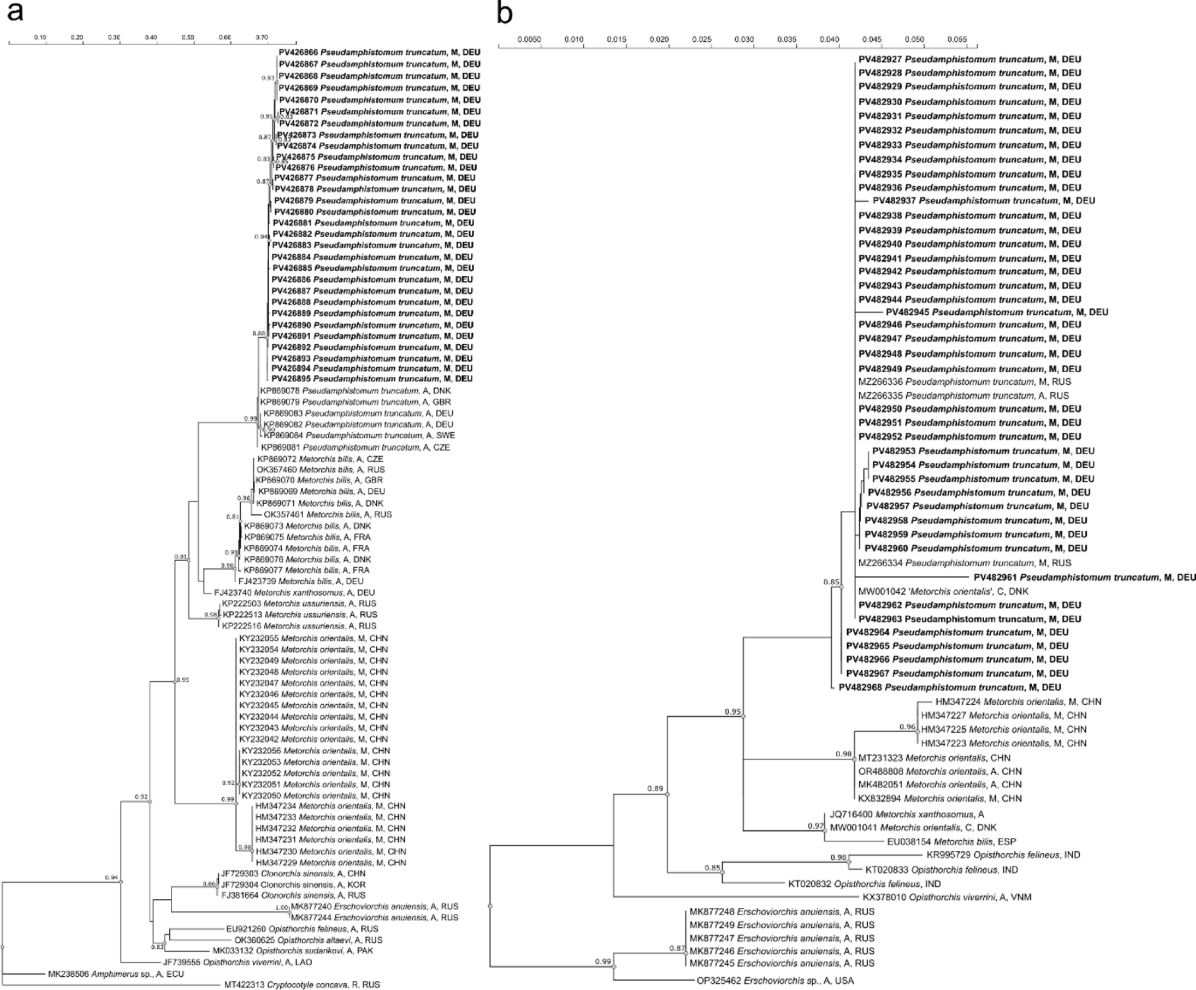
Dendrogram based on maximum-likelihood analysis of (**a**) partial cox1 sequences and (**b**) ITS1 sequences of the opisthorchiid isolates obtained in this study (bold) and sequences obtained from GenBank. Only bootstrap values > 80% are shown. R, redia; C, cercaria; M, metacercaria; A, adult; CHN, China; CZE, Czech Republic; DEU, Germany; DNK, Denmark; ECU, Ecuador; ESP, Spain; GBR, United Kingdom; IND, India; KOR, Korea; LAO, Laos; FRA, France; PAK, Pakistan; SWE, Sweden; RUS, Russia; USA, United States of America; VNM, Vietnam.

**Fig. 3:**
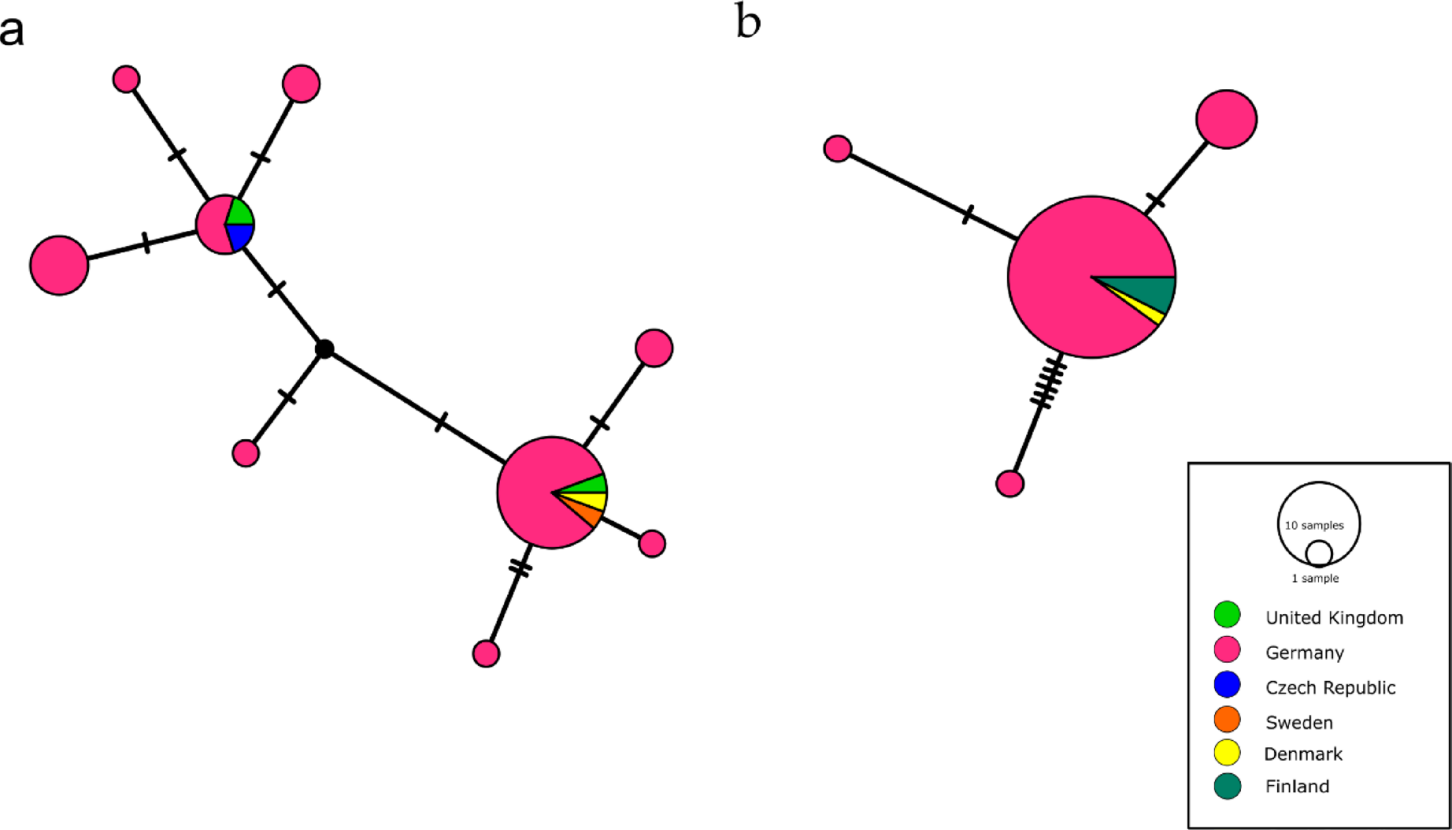
TCS-haplotype network based on (**a**) cox1 and (**b**) ITS1 sequences of the opisthorchiid isolates obtained in this study.

The phylogenetic analysis based on cox1 sequences retrieved a well-supported clade (Bootstrap support 99%) including all the samples from this study and isolates of *P. truncatum* deposited in GenBank (Fig. 2a). The pairwise distances of the cox1 sequences obtained in this study varied between 0% and 2.4%, and between 0% and 3.9% for all *P. truncatum* sequences. The TCS haplotype network analysis of the cox1 dataset including the haplotypes of Sherrard-Smith^31^, identified nine haplotypes (Fig. 3a). Four haplotypes were found only once in German samples, three of them in River Spree and one in Lake Müggelsee. More than one haplotype was detected in three tench from River Spree, the locality with the highest number of haplotypes. Specimens from Germany, United Kingdom, Sweden and Denmark shared the most frequently detected haplotype in terms of samples. The cox1 dataset had 10 segregating sites, 5 of which were parsimony informative. The nucleotide diversity (Pi) was 0.00143 and Tajima’s D was − 2.63323 (*p* < 0.05).

Forty-two partial ITS1 sequences were generated. The closest matches to our isolates were two sequences deposited in GenBank under the names *Pseudamphistomum truncatum* (MZ266334) and *Metorchis orientalis* (MW001042).

The dataset for phylogenetic analysis based on ITS1 included our sequences and additional opisthorchiid isolates available from GenBank (Supplementary table S1). The final alignment comprised 684 bp. A cluster with moderate statistical support (bootstrap 85%) grouped all our isolates, *P. truncatum* (MZ266334- MZ266336) and ‘*M. orientalis*’ (MW001042) (Fig. 2b). The pairwise distances of the ITS sequences of our opisthorchiids and the closest matches in the GenBank ranged between 0.00% and 1.69%.

The TCS-haplotype network analysis of ITS1 (Fig. 3b) distinguished four haplotypes among *P. truncatum*, the most dominant haplotype was shared by samples from Germany, Denmark and Finland; three haplotypes were found only in Germany, two of them detected in single individuals. The dataset contained eight segregating sites, one parsimony informative. The nucleotide diversity (Pi) was low 0.00098, and Tajima’s D was − 2.0075 (*p* < 0.05).

### Morphological description of diplostomid metacercariae

Most diplostomid metacercariae were found free, not encapsulated in the muscle. The few specimens found encysted had a granular, round, single-layered, and extremely fragile cyst (Fig. 1d). The body of the metacercaria had a pyriform shape and was divided in two distinct parts (Fig. 1e). The tegument was covered by small papillae. The forebody was round and ventrally concave pseudosuckers were located at both sides of the subterminal oral sucker. The trilobated holdfast organ was situated ventrally in the middle of the forebody. The ventral sucker located near the anterior margin of the holdfast organ was slightly bigger than the oral sucker (U = 8.5, *p* = 0.01). In some cases, the ventral sucker was partially or totally covered by the holdfast organ and not visible. The hindbody consisted of a cylindrical to subtriangular prolongation, widest at the junction with the forebody. The intestinal caeca reached the posterior part of the hindbody. Morphometric data of metacercariae are provided in Table 2.

### Molecular characterization of diplostomid metacercariae

**Fig. 4:**
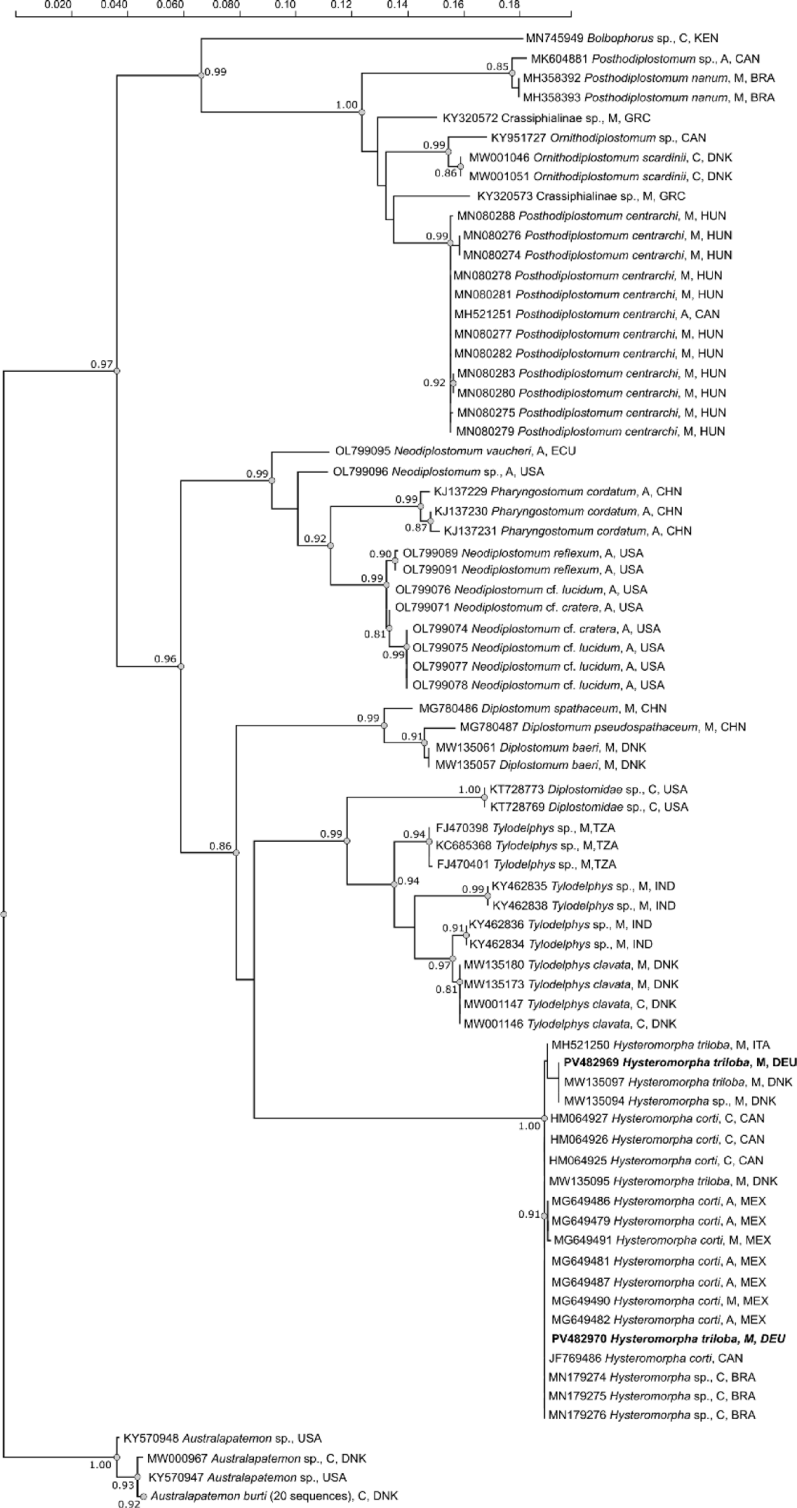
Dendrogram based on maximum-likelihood analysis of ITS sequences of the diplostomid isolates of this study (bold) and sequences obtained from GenBank. Only bootstrap values > 80% are shown. C, cercaria; M, metacercaria; A, adult; BRA, Brazil; CAN, Canada; CHN, China; DEU, Germany; DNK, Denmark; ECU, Ecuador; GRC, Greece; HUN, Hungary; IND, India; KEN, Kenya; MEX, Mexico; TZA, Tanzania; USA, United States of America.

Two sequences of the complete ITS1–5.8 S–ITS2 region were highly similar (> 99%) to 14 isolates of *Hysteromorpha triloba* and *Hysteromorpha* sp. from GenBank (MG649479, MG649481, MG649482, MG649486, MG649487, MG649490, MG649491, MH521250, MN179274-MN179276, MW135094, MW135095, MW135097) and four isolates assigned to *Hysteromorpha corti* (JF769486, HM064925-HM064927), although identified as *H. triloba* in their respective references^32,33^. These sequences were co-analyzed with other diplostomid sequences available in GenBank, comprising a total of 94 sequences (Supplementary table S1). The alignment was 1063 bp long. In the ML tree, both isolates clustered with sequences of *H. triloba*,* H. corti and Hysteromorpha* sp. obtained from GenBank (Fig. 4). in a clade with strong bootstrap support (100%). The pairwise distances among *Hysteromorpha* in the dataset varied from 0 to 0.3%.

### Morphological description of cyathocotylid metacercariae

The cysts of cyathocotylid metacercariae were round, the most internal layer was thinner than the others, and almost completely occupied by the metacercaria (Fig. 1f). The body of the metacercaria was sub-oval in shape, narrow anteriorly (Fig. 1g). The pharynx was slightly smaller than the subterminal oral sucker, the esophagus rather short, and the intestinal branches extended to the posterior end of the body (Fig. 1g). The body had a shallow ventral concavity in the posterior half of the body, where the round holdfast organ was located. The ventral sucker, situated anteriorly to the holdfast organ, a little behind the center of the body (Fig. 1g), was almost half the size of the oral sucker (U = 193.5, *p* < 0.001). The ventral sucker was not visible in all individuals. The channels of the secondary excretory system covered almost entirely the body and masked most of the organs. Small round granules were visible within the tubules. Morphometric data of metacercariae are given in Table 2.

### Molecular characterization of cyathocotylid metacercariae

**Fig. 5:**
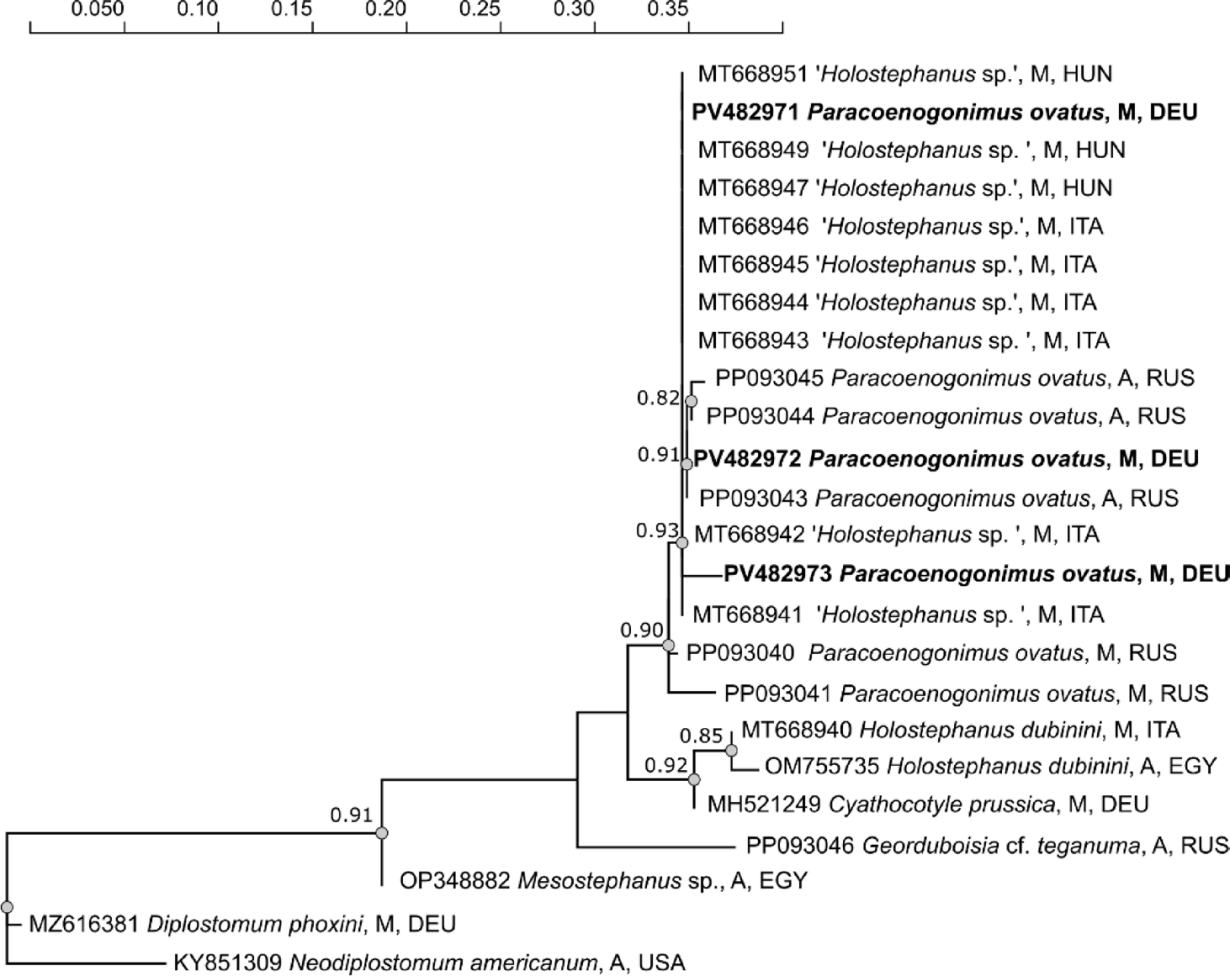
Dendrogram based on maximum-likelihood analysis of ITS sequences of the cyathocotylid isolates of this study (bold) and sequences obtained from GenBank. Only bootstrap values > 80% are shown. M, metacercaria; A, adult; DEU, Germany; EGY, Egypt; HUN, Hungary; ITA, Italy; RUS, Russia; USA, United States of America.

Sequences of the complete ITS1–5.8S–ITS2 region of three specimens were obtained. The closest matches found in GenBank corresponded to isolates designated as *Holostephanus* sp. (MT668941 to MT668949 and MT668951) or therein assigned as *Paracoenogonimus ovatus* (PP093043 and PP093044) with similarity values ranging from 99.5 to 100%. The phylogenetic analysis involved 24 sequences (Supplementary table S1) and the alignment comprised 418 bp. A relatively well-supported clade (90%) included cyathocotylid metacercariae from tench, together with metacercaria assigned as ‘*Holostephanus* sp.’ and adults deposited as *Paracoenogonimus ovatus* in GenBank (Fig. 5).

Two of our specimens (PV428971 and PV428972) were almost identical to metacercaria of *Holostephanus* sp. and adults of *P. ovatus* (PP093043 and PP093044) with distances between 0% and 0.4%. The genetic divergence between these isolates and our specimen PV428973 varied between 1.4% and 1.9%, and two metacercaria identified as *P. ovatus* (PP093040 and PP093041) differed between 1.2 and 3.4% from our samples PV428971-973.

## Discussion

Previous cases of human infections from Italy showed that the consumption of raw or marinated tench fillets can be the cause of opisthorchiasis^27,28,34^. Furthermore, in the endemic areas of central Italy, tench serves as a particularly suitable host for *Opisthorchis felineus*, with a proven prevalence of almost 90% in the Bolsena and Bracciano lakes^35^. Although in the past opisthorchiid trematodes have been recorded in north-eastern Germany with high prevalence in cats and red foxes as definitive hosts^19,21,23^ and various cyprinid fish species as second intermediate hosts^19,20,22^, tench, which are popular as food fish, have not yet been examined for muscle metacercariae in Germany. Based on both morphological and molecular identification, we have detected metacercariae of the Opisthorchiidae, Cyathocotylidae and Diplostomidae families in muscle tissue of tench.

An unequivocal identification of opisthorchiid metacercariae only based on morphometrics is challenging, as the metacercariae of the European liver flukes *O. felineus*,* M*,* bilis*,* M. xanthosomus* and *P. truncatum* are morphologically very similar, differing mainly in their size, while size ranges of these species may even overlap, with *P. truncatum* regarded as the largest species^20^. Based solely on the comparison of the morphometric data of the opisthorchiid metacercariae isolated from tench in this study with data from the above-mentioned references, a possible co-infection with *P. truncatum* and *O. felineus* could be assumed, and the presence of the smaller *Metorchis* spp. could not be excluded. Moreover, all of them have been reported from Germany^20,22^. However, in the interspecific phylogenetic assignment based on cox1, all sequences generated from our opisthorchiid metacercariae were placed in a well-supported clade with adult specimens of *Pseudamphistomum truncatum*^31^, indicating their conspecificity and excluding the other morphologically similar species. The values of distances based on cox1 sequences were slightly higher than the range of genetic distances within species of the family Opisthorchiidae^36^, but lower than its interspecific divergence^37^, consistent with variation in other trematode families^38^. In the phylogenetic tree based on ITS1, a cluster included our sequences and a metacercaria isolate of *P. truncatum* (MZ266334) from Russia, and a cercaria of *M. orientalis* (MW001042) collected from the lymnaeid snail *Stagnicola palustris* in freshwater ponds in Denmark^39^. A probable misidentification by Duan et al.^39^ was suggested by Sokolov et al.^30^ taking into account further phylogenetic analysis, geographical distribution and because lymnaeid snails are not known as intermediate hosts of opisthorchiid trematodes.

The genetic divergence of the ITS1 sequences was similar or slightly higher compared with the intraspecific one within Opisthorchiidae^36,40,41^ or other trematode families^38^, without reaching interspecific values^40^. Irrespective of the large morphological variation observed in our metacerariae from tench and beyond those previously reported for the species^42–45^, the relatively low genetic distances below interspecific limits and their clustering with *P. truncatum* in both phylogenetic trees, particularly with adult specimen of *P. truncatum*^31^ strongly support their conspecificity with *P. truncatum*.

Combined morphological and molecular analyses of the diplostomid metacercariae from tench led to an unambiguous identification as *Hysteromorpha triloba sensu lato*. For a long time, *H. triloba* was considered cosmopolitan, until it was recently subdivided into the Palaearctic *H. triloba* and the Nearctic *H. corti*^46^ taxon. That the sequences of our two *Hysteromorpha* isolates from Germany cluster together with those from Brazil, Mexico, Canada, Italy and Denmark reflects the low variability caused by high evolutionary conservation of the rDNA, as already referred to by Locke et al.^46^. Anyhow, according to the biogeographical subdivision, individuals collected in Germany are to be designated as *H. triloba*. Their morphology and morphometric characters were similar to previous descriptions of *H. triloba*^46–48^, and molecular analysis confirmed this identification. Likewise, the low pairwise distances among all sequences of *Hysteromorpha* included in the dataset agree with reported intraspecific variation^46,49,50^.

Cyathocotylids isolated from tench agreed with the original description of metacercaria of *Paracoenogonimus ovatus*^51^, and the body size of our largest specimens corresponded approximately to that of the specimen described therein. The ventral sucker was not visible in all specimens isolated from tench, suggesting different developmental degrees of the metacercariae. According to Komiya^52^, the ventral sucker is clearly visible only in older metacercaria, while it is not developed in metacercariae younger than five weeks. In the phylogenetic tree of ITS sequences, our cyathocotylid metacercariae clustered with metacercariae assigned as *Holostephanus* sp. (Hungary and Italy), and adults and a metacercaria, identified as *Paracoenogonimus ovatus* (Russia). The smaller body size of *Holostephanus* spp. and morphological characteristics distinguishing the genera *Paracoenogonimus* (subfamily Prohemistominae) and *Holostephanus* (subfamily Cyathocotylinae)^47,53^ suggest that the cyathocotylid metacercariae isolated from tench belong to *P. ovatus*. These include Brandes organ, which in *Paracoenogonimus* is smaller in relation to the body size and located in the posterior part of the body, and the position of the ventral sucker in the middle of the body, at a clear distance from the intestinal furcation. Furthermore, the low genetic divergence between our cyathocotylid metacercariae, those from Hungarian and Italian fishes^54^ and adult specimens of *P. ovatus* of Sokolov et al.^55^ further support that all belong unequivocally to *P. ovatus*. However, additional molecular data of the species along its distribution range and from different host species are needed to address the question whether the range of variation observed between some specimens still falls within intraspecific levels, especially as *P. ovatus* may represent a species complex^55,56^.

The occurrence of *P. truncatum* in tench constitutes a new host record in Germany. In Europe, hitherto metacercariae of two opisthorchiid species have been detected in tench: *P. truncatum* in UK^31^ and Italy^9^, and *O. felineus* in Italy^35^. The prevalences of *P. truncatum* metacercariae in tench from natural waterbodies from 46 to 100% were similar to prevalences reported for roach in the Gulf of Finland with 46%^57^ and in the River Shannon in Ireland with 89%^58^ and higher than prevalences of opisthorchiids infecting roach from the Berlin area (River Havel, Lake Müggelsee and Teltowkanal) with values of 1.3% for *P. truncatum* and 28.8% for metacercariae of the “*O. felineus-M. bilis*” type^22^.

*Paracoenogonimus ovatus*, which was originally described from metacercariae collected in Germany^52^, is known as a parasite of tench^54^, and co-infections with metacercariae of the *P. truncatum* and *P. ovatus* were also observed by Simakova et al.^59^ in the middle Ob River basin. Tench harbouring metacercaria of *P. ovatus* have been reported in Poland^60,61^ with a prevalence of 19%^60^. Similar prevalences to those of our study have been observed in roach and bleak in natural waters of Poland^62^ and in the Mykolaiv Region of Ukraine^63^. High prevalences of metacercariae of *P. ovatus* (classified as Cyathocotylidae gen. sp.) were found in carp from fish farms in Hungary^54^.

Metacercariae of *H*. *triloba* have been previously reported in tench and other cyprinids in Germany^20,64^. The only known final host for *H. triloba* in Europe is the black cormorant (*Phalacrocorax carbo)*^42,47^, and it is not suspected of infecting mammals. The different prevalences of *H. triloba* in tench probably reflect different degrees of occurrences of cormorants, suggesting larger populations around Lake Müggelsee and the fish farm in Brandenburg.

The zoonotic potential of *P. truncatum* detected in tench may be inferred from several reports that show fish-eating mammals as their final hosts^44,58,65^, and that it can also infect humans^66^. In contrast, the zoonotic potential of *P. ovatus* is discussed controversially. Several publications indicate possible infections not only of avian hosts but also in humans^60,61^. Mice and laboratory rats have also been successfully infected experimentally^51,62^. However, the experiments conducted by Sándor et al.^67^ with metacercariae of *P. ovatus* (misidentified as *Holostephanus* sp.) were unsuccessful, which led Sokolov et al.^55^ to suggest that *P. ovatus* found in mammals and birds could probably belong to different species. This latter example shows how important unequivocal taxonomic identification is for human health assessment.

The presence of common haplotypes of *P. truncatum* in different European countries suggests gene flow between populations, possibly promoted by the high dispersal capacities of mobile hosts across Europe and are corresponding to low nucleotide diversity^68^. The significant negative values of Tajima’s D indicate an excess of rare genetic variants that could be associated with population expansion, which is in agreement with the emergence of *P. truncatum* in various final hosts throughout Europe^44,58,65^. There are no discernible limits for the spread of *P. truncatum*, as its snail and cyprinid intermediate hosts are widely distributed in Europe^31^.

The high prevalence of *P. truncatum* in tench, especially from natural waters, clearly shows that zoonotic trematodes can have a serious impact on food safety and public health in Central Europe. In addition, the zoonotic potential of the widespread *P. ovatus* cannot yet be ruled out. Our study indicates that *P. truncatum* is more prevalent in natural waters than in aquaculture ponds. However, it also shows that the presence of zoonotic opisthorchiid trematodes cannot be excluded in pond culture. So far, the risk of opisthorchiasis in Germany has been regarded as low because the consumption of raw or undercooked cyprinids has no tradition here^20^, and reported cases in humans are rare, with the most recent case in 2009^69^. Compared to the main species rainbow trout and carp, tench plays only a minor role in German aquaculture production^70^. However, as assumed by Schuster et al.^20^, anglers in particular could run the risk of becoming infected with opisthorchiids, e.g. by eating cold-smoked fish, especially as they target and consume also other fish species such as roach (*Rutilus rutilus*), bream (*Abramis brama*) and rudd (*Scardinius erythrophthalmus*)^26^, which play no major role in the fish trade, and can also serve as fish hosts for the parasite^20,42,43^. Another group with an increased risk of opisthorchiasis are immigrants, especially from eastern countries with different eating traditions^20^. In addition, repeated outbreaks of opisthorchiasis in Italy, which are attributed to *Opisthorchis felineus*, clearly show the risk of infection through the consumption of corresponding fish delicacies in a restaurant^27,28,34,71^ and revealed the possible fish substitutions using tench as whitefish for food fraud^34^. The general lack of research on zoonotic parasites specifically in European freshwater fish, and the trend towards consumption of raw or semi-marinated fish contrasts with a lack of surveillance data on fish-borne zoonotic parasites^72^. It should be noted here that more parasites in freshwater fish than those mentioned in this article have zoonotic potential, including *Clinostomum complanatum* (Trematoda), *Contracaecum rudolphii*, *Eustrongylides excisus* (Nematoda), *Dibothriocephalus latus* (Cestoda)^73^. Our study shows that the parasites are present, and it will only depend on the spread of both traditional and new trends in food preparation whether infective larvae are ingested, and human infections may occur. In conclusion, more and improved surveillance across more freshwater fish species and in multiple waterbodies is a requirement of zoonotic disease prevention in Central Europe.

## Materials and methods

### Sample collection

**Fig. 6:**
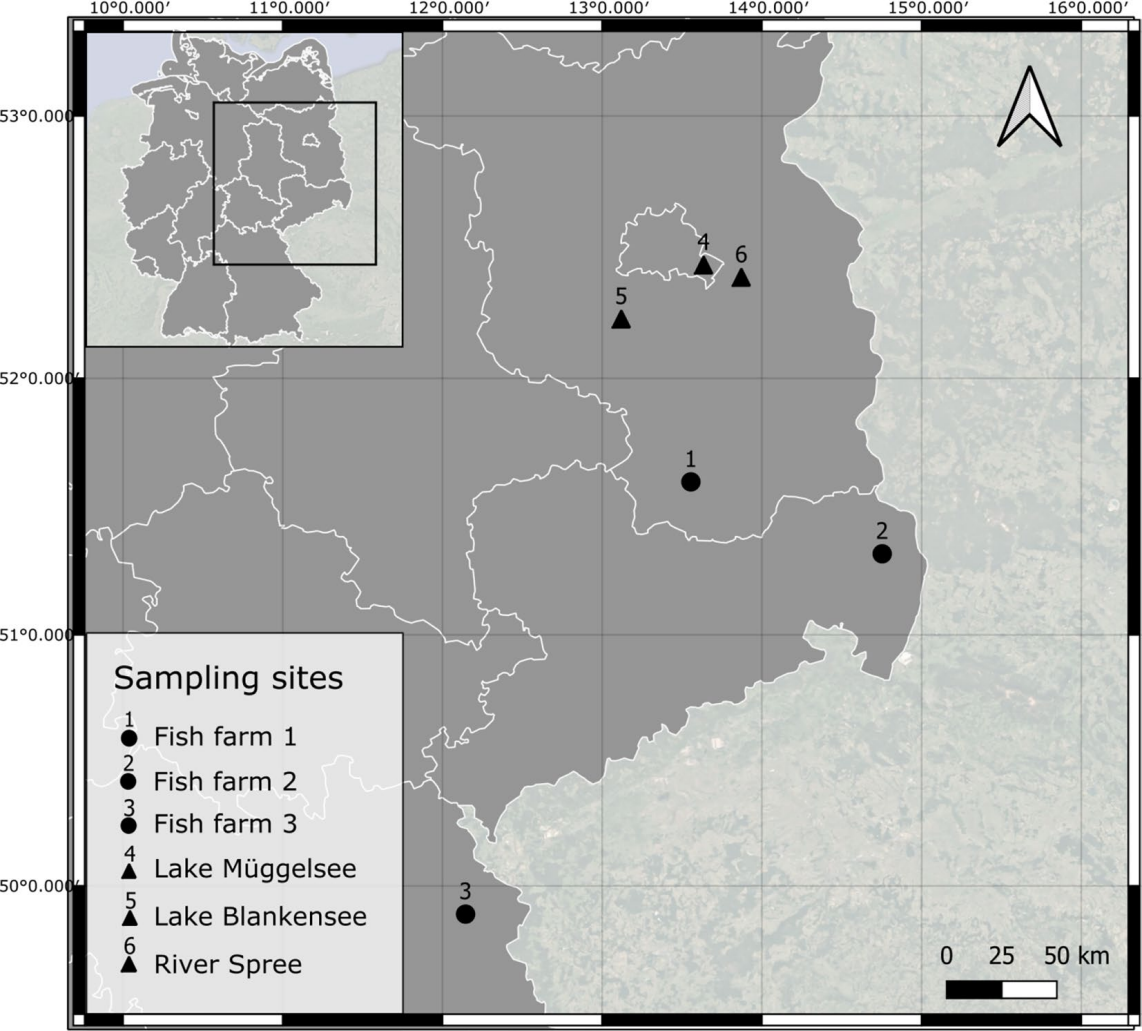
Location of the sampled natural waters (triangles) and fish farms (circles) in Germany. Figure was created using QGIS version 3.10.6 (https://qgis.org/). Germany’s administrative map VK2500 was obtained from GeoBasis-DE under the license “dl-de/by-2-0” (www.govdata.de/dl-de/by-2-0).

From March to September 2022, tench were obtained from three fish farms (1–3) located in Brandenburg (1), Saxony (2), and Bavaria (3) and from three natural waterbodies (4–6) in Berlin and Brandenburg, namely Lake Müggelsee (4), Lake Blankensee (5), and River Spree (6) near Mönchswinkel (Fig. 6).

Weight and total length of the fish were measured prior to dissection, to the nearest of 1 g or 1 mm, respectively. Fish fillets were examined individually through the compression method according to Waikagul & Thaenkham^1^. Cysts were gently isolated using dissection needles and placed into Petri dishes with 0.7% saline. Living metacercariae within cysts and after their mechanical excystment were individually and without pressure examined on microscopic slides with a drop of 0.7% saline and photographed at 50x, 100x and 200x magnification. Morphological and morphometric characters of the metacercariae including size, shape and number of layers of the cyst, length and width of the body, size of oral and ventral suckers, and of other taxonomically relevant structures were recorded. All measurements were taken to an accuracy of 1 μm.

The morphological identification of the metacercariae followed the taxonomic keys of Bykhovskaya-Pavlovskaya et al.^42^, Bauer^43^, and Niewiadomska^47^. The identification of individual specimens was followed by their preservation in 96% ethanol for further molecular analyses. The selection criteria of metacercaria specimens used in molecular analyses included a relative uncertainty level in preliminary identification based on morphology, the representation of intraspecific morphological variation and different sampling locations.

### DNA extraction

DNA was extracted from whole individual excysted metacercaria using the QIAamp DNA mini kit (Qiagen, Hilden, Germany) with slight modifications to the manufacturer’s protocol as follows: 90 µl of Buffer ATL and 10 µl of Proteinase K were added to the tubes, mixed, and incubated at 56 °C for 2 h. The lysis was followed by the addition of 100 µl of Buffer AL and incubation for 10 min at 70 °C. One hundred microliters of molecular grade ethanol were incorporated to the mixture and pipetted onto the QIAamp Mini spin column placed in a 2 ml collection tube and centrifuged (8000 rpm, 1 min). After centrifugation, the spin column was placed into a new 2 ml collection tube and the flow-through discarded. The same procedure was repeated in the next two steps after adding 500 µl of Buffer AW1 (8000 rpm, 1 min) and subsequently 500 µl of Buffer AW2 (14000 rpm, 3 min). Finally, 35 µl of Buffer AE was added to the tube, incubated at 37 °C for 5 min and centrifuged (10000 rpm, 1 min). The concentration of DNA yielded was estimated in a microvolume spectrophotometer (NanoDrop ND-1000, Marshall Scientific, Hampton, USA). The DNA samples were stored at −20 °C until PCRs were run.

### PCR amplification

All PCR-amplifications were conducted on a Mastercycler Nexus GSX1 (Eppendorf, Hamburg, Germany). Negative and positive controls were included in each amplification run.

New degenerate primers targeting an 837 bp fragment of the mitochondrial cox1 region of opisthorchiids were designed using available sequences of the family Opisthorchiidae from the GenBank database, namely the primers cox1_opist_F (5´-CDATGGATCAYAAGCGTATAGG-3´) and cox1_opist_R (5´- TGATGAGCTCAAACMACMCT-3´). The PCR had a total volume of 25 µl and included 5 µL 5x PCR AllTaq Buffer (Qiagen), 0.5 µL dNTP, 1.25 µL of each primer, 0.5 µL AllTaq Polymerase (Qiagen), 6 µl of DNA template and 10.5 µL H_2_O. PCR conditions were 93 °C for 3 min, 40 cycles of 93 °C for 20 s, 55 °C for 30 s, and 72 °C for 1 min, with a final extension at 72 °C for 5 min. The nuclear ITS1-region of opisthorchiids was amplified with the forward primer (5´-CAAGGTTTCCGTAGGTGA-3´) and reverse primer (5-CTGCGTTCTTCATCGACAC-3´)^41^. The PCR-reactions included 2.5 µL 5x PCR AllTaq Buffer (Qiagen), 1.25 µL MgCl_2_, 0.5 µL dNTP, 1.25 µL of each primer, 0.5 µL AllTaq Polymerase (Qiagen), 6 µl of DNA template, and 11.75 µL H_2_O for a total volume of 25 µl. PCR conditions were 95 °C for 2 min, 40 cycles of 94 °C for 1 min, 58 °C for 45 s, and 72 °C for 45 s, with a final extension at 72 °C for 5 min.

A segment of the rDNA (1065 bp) including the complete ITS1–5.8S–ITS2 region of diplostomids and cyathocotylids was amplified with the primers D1 (5′-AGGAATTCCTGG TAAGTGCAAG-3′) and D2 (5′- CGTTACTGAGGGAATCCTGGT-3′)^74^. The PCR had a total volume of 25 µl and included 5 µL 5x PCR AllTaq Buffer (Qiagen), 3 µL MgCl2, 1 µL dNTP, 1.25 µL of each primer, 0.5 µL AllTaq Polymerase (Qiagen), 6 µl of DNA template, and 7 µL H_2_O – PCR grade. PCR conditions were 94 °C for 2 min, 40 cycles of 94 °C for 1 min, 56 °C for 1 min, and 72 °C for 2 min, with a final extension at 72 °C for 5 min.

### Sequence generation, alignment, and species assignment

PCR products were visualized using electrophoresis with 1.5% TAE agarose gels stained with ethidium bromide. Amplified PCR-products were sent to Eurofins Genomics (Ebersberg, Germany) for purification and Sanger-sequencing. The electropherograms of the generated sequences were evaluated and their ends trimmed using Finch TV v1.4.0 (Geospiza, Inc.; Seattle, WA, USA). Consensus sequences were constructed using SeaView v. 5 software^75^.

In order to corroborate preliminary morphological identification, a first assessment with searches of the nucleotide sequences generated using the Basic Alignment Search Tools (BLAST) of the National Center for Biotechnology Information (NCBI) (https://www.ncbi.nlm.nih.gov/) was complemented with phylogenetic analyses, per family and molecular marker, to verify the position of the sequenced individuals in the phylogenetic tree. Alignments of the generated sequences were built separately using MUSCLE^76^ as implemented in MEGA 11.0^77^ with similar sequences retrieved by BLAST searches or previous phylogenetic studies of the families (Supplementary table S1). ML analyses were performed using PhyML3.0^78^ on the ATGC bioinformatics portal (http://www.atgc-montpellier.fr/). The Bayesian Information Criterion was used to determine the best-fit nucleotide substitution model for each dataset, namely the HKY85 + G + I for the alignment of Opisthorchiidae (cox1), HKY85 + G for Opisthorchiidae (ITS1), GTR + G for Diplostomidae (ITS), and K80 + G for Cyathocotylidae (ITS). Pairwise genetic divergence within taxon was determined using uncorrected distances (p-distance) calculated in MEGA 11.0 with default settings. The program PopArt (Population Analysis with Reticulate Trees) v1.7^79^ was used to construct unrooted statistical parsimony TCS haplotype networks and to estimate the number of segregating sites, the number of parsimony informative sites, nucleotide diversity (Pi), and the Tajima’s D test.

### Statistical analyses

The Kruskal-Wallis H-test followed by Dunn’s multiple comparison tests was applied to test for significant differences between localities in the mass of tench and infection intensities of metacercariae. The differences between localities in prevalence of infection were evaluated using pairwise Chi-square tests with Bonferroni correction for multiple comparisons. For all analyses, significance was accepted when *P* ≤ 0.05. Confidence intervals were calculated according to Newcombe & Soto^80^. Statistical analyses were performed using R Statistical Software (v 4.4.1)^81^.

## Electronic supplementary material

Below is the link to the electronic supplementary material.

Supplementary Material 1

## Data Availability

Sequences were submitted to GenBank under the accession numbers PV426866-PV426895 and PV482927-PV482968 (*Pseudamphistomum truncatum*); PV482969-PV482970 (*Hysteromorpha triloba*), and PV482971-PV482973 (*Paracoenogonimus ovatus*). All raw sampling and measurement data generated are available from the corresponding author on reasonable request.
