# Supplementary material for: Morphological and molecular assessment of muscle metacercariae infecting tench Tinca tinca from fish farms and wild populations in Germany
Source: Sci Rep. 2025 Jul 3;15:23700. doi: 10.1038/s41598-025-09396-y (PMC12229617; doi:10.1038/s41598-025-09396-y)
Supplement: Supplementary file 1 — Supplementary Material 1 [file 41598_2025_9396_MOESM1_ESM.docx]

| **Supplementary Table S1. List of GenBank Accession numbers, species, life stage, host, geographic origin and references of the sequences included in molecular analyses.** | | | | | |
| --- | --- | --- | --- | --- | --- |
| Accession number/Sample ID | Species | Stage | Host | Geographic origin | Reference |
| **COX1 Opisthorchiidae** | | | | | |
| MT422313 | *Cryptocotyle concava* | Redia | *Peringia ulvae* | Russia | Gonchar 2020 (Direct submission) |
| MK238506 | *Amphimerus* sp. | Adult |  | Ecuador | Ma et al. 2019 |
| OK360625 | *Opisthorchis altaevi* | Adult | *Ardea purpurea* | Russia | Sokolov et al. 2022 |
| JF739555 | *Opisthorchis viverrini* | Adult | *Felis catus* | Laos | Cai et al. 2012 |
| MK033132 | *Opisthorchis sudarikovi* | Adult | *Egretta garzetta* | Pakistan | Suleman et al. 2019 |
| EU921260 | *Opisthorchis felineus* | Adult | *Felis catus* | Russia | Shekhovtsov et al. 2010 |
| MK877240, MK877244 | *Erschoviorchis anuiensis* | Adult | duckling | Russia, Amur River | Tatonova et al. 2020 |
| FJ381664 | *Clonorchis sinensis* | Adult | *Mesocricetus auratus* (experimental host) | Russia | Shekhovtsov et al. 2010 |
| JF729303  JF729304 | *Clonorchis sinensis* | Adult | *Felis catus* | China  Korea | Cai et al. 2012 |
| HM347229, HM34730, HM347231, HM347232, HM347233, HM347234 | *Metorchis orientalis* | Metacercaria | *Pseudorasbora parva* | China | Ai et al. 2010 |
| KY232042 - KY232056 | *Metorchis orientalis* | Metacercaria | *Pseudorasbora parva* | China | Gao 2016 (Direct submission) |
| KP222503, KP222513, KP222516 | *Metorchis ussuriensis* | Adult | duck | Russia, Magdikovoe | Besprozvannykh et al. 2019 |
| FJ423740 | *Metorchis xanthosomus* | Adult |  | Germany | Krone et al. 2008 (Direct submission) |
| FJ423739 | *Metorchis bilis* | Adult |  | Germany | Krone et al. 2008 (Direct submission) |
| KP869069  KP869070  KP869071, KP869073,  KP869076  KP869072  KP869074, KP869075, KP869077 | *Metorchis bilis*  (syn. *M. albidus*) | Adult | *Lutra lutra*  *Neovison vison* | Germany  United Kingdom  Denmark  Czech Republic  France | Sherrard-Smith et al. 2016 |
| OK357460, OK357461 | *Metorchis bilis* | Adult | *Milvus migrans* | Russia | Sokolov et al. 2022 |
| KP869078  KP869079, KP869084  KP869081  KP869082  KP869083 | *Pseudamphistomum truncatum* | Adult | *Lutra lutra*  *Neovison vison* | Denmark  United Kingdom  Sweden  Czech Republic  Germany | Sherrard-Smith et al. 2016 |
| PV426866 - PV426895 | *Pseudamphistomum truncatum* | Metacercaria | *Tinca tinca* | Germany | This study |
| **ITS1 Opisthorchiidae** | | | | | |
| OP325462 | *Erschoviorchis* sp. | Adult | *Haliaeetus leucocephalus* | USA | McDermott et al. 2022 (Direct submission) |
| MK877245 - MK877249 | *Erschoviorchis anuiensis* | Adult | *Cairina moschata* (experimental host) | Russia, Amur River | Tatonova et al. 2020 |
| KX378010 | *Opisthorchis viverrini* | Adult | Felis catus | Vietnam | Hung et al. 2016 (Direct submission) |
| KT020832, KT020833,  KR995729 | *Opisthorchis felineus* | Not specified | Not specified | India | Gamit et al. 2015 (Direct submission) |
| EU038154 | *Metorchis bilis* | Not specified | Not specified | Spain | Kang et al. 2008 |
| MW001041 | *Metorchis orientalis* | Cercaria | *Stagnicola palustris* | Denmark, Lake Lyngby | Duan et al. 2021b |
| KX832894 | *Metorchis orientalis* | Metacercaria | Not specified | China | Zhang et al. 2016 (Direct submission) |
| MK482051 | *Metorchis orientalis* | Adult | Not specified | China | Qiu et al. 2019 (Direct submission) |
| OR488808 | *Metorchis orientalis* | Adult | Anatidae | China | Li 2023 (Direct submission) |
| MT231323 | *Metorchis orientalis* | Not specified | Not specified | China | Wang et al. 2020 |
| HM347223-HM347225, HM347227 | *Metorchis orientalis* | Metacercariae | *Pseudorasbora parva* | China | Ai et al. 2010 |
| MW001042 | *‘Metorchis orientalis‘* | Cercaria | *Stagnicola palustris* | Denmark, Lake Lyngby | Duan et al. 2021b |
| JQ716400 | *Metorchis xanthosomus* | Adult | *Circus aeruginosus* | Not specified | Hotzel et al. 2012 (Direct submission) |
| MZ266334, MZ266336  MZ266335 | *Pseudamphistomum truncatum* | Metacercaria  Adult | *Rutilus rutilus*  *Vulpes vulpes* | Russia  Finland | Kathokin et al. 2021 (Direct submission) |
| PV482927-PV482968 | *Pseudamphistomum truncatum* | Metacercaria | *Tinca tinca* | Germany | This study |
| **ITS Diplostomidae** | | | | | |
| OL799096 | *Neodiplostomum* sp*.* | Adult | *Bubo virginianus* | USA, North Dakota | Achatz et al. 2022 |
| OL799095 | *Neodiplostomum vaucheri* | Adult | *Trachops cirrhosus* | Ecuador | Achatz et al. 2022 |
| OL799091  OL799089 | *Neodiplostomum reflexum* | Adult | *Strix varia*  *Buteo jamaicensis* | USA, Mississippi  USA, North Dakota | Achatz et al. 2022 |
| OL799075  OL799076  OL799077, OL799078 | *Neodiplostomum* cf. *lucidum* | Adult | *Didelphis virginiana*  *Lithobates catesbeianus* | USA, Arkansas  USA, Nebraska  USA, Mississippi | Achatz et al. 2022 |
| OL799071  OL799074 | *Neodiplostomum* cf. *cratera* | Adult | *Didelphis virginiana*  *Neogale vison* | USA, Mississippi  USA, Minnesota | Achatz et al. 2022 |
| MW135173  MW135180 | *Tylodelphys clavata* | Metacercaria | *Rutilus rutilus*  *Perca fluviatilis* | Denmark, Lake Bromme lilleso | Duan et al. 2021a |
| MW001146  MW001147 | *Tylodelphys clavata* | Cercaria | *Ampullaceana balthica* | Denmark, Lake Brommelille  Denmark, Lake Bagsvaerd | Duan et al. 2021b |
| MW135095, MW135097 | *Hysteromorpha triloba* | Metacercaria | *Rutilus rutilus* | Denmark, Lake Bromme lilleso | Duan et al. 2021a |
| MH521250 | *Hysteromorpha triloba* | Metacercaria | *Squalius cephalus* | Italy, Bidente River | Locke et al. 2018 |
| PV482969, PV482970 | *Hysteromorpha triloba* | Metacercaria | *Tinca tinca* | Germany | This study |
| MG649490, MG649491  MG649486, MG649487  MG649479 - MG649482 | *Hysteromorpha triloba* | Metacercaria  Adult | *Astyanax mexicanus*  *Nannopterum brasilianus* | Mexico, San Luis Potosi  Mexico, Veracruz  Mexico, Chiapas | Sereno-Uribe et al. 2018 |
| JF769486 | *Hysteromorpha corti* |  |  | Canada | Locke et al. 2011 |
| HM064925 - HM064927 | *Hysteromorpha corti* | Metacercaria | *Catostomus commersonii* | Canada | Locke et al. 2010 |
| MW135094 | *Hysteromorpha* sp. | Metacercaria | *Rutilus rutilus* | Denmark: Lake Bromme lilleso | Duan et al. 2021a |
| MN179274 - MN179276 | *Hysteromorpha* sp. | Cercaria | *Biomphalaria straminea* | Brazil: Minas Gerais | Lopez-Hernandez et al. 2019 |
| MW135057, MW135061 | *Diplostomum baeri* | Metacercaria | *Perca fluviatilis* | Denmark: Lake Bromme lilleso | Duan et al. 2021a |
| MW001046, MW001051 | *Ornithodiplostomum scardinii* | Cercaria | *Ampullaceana balthica* | Denmark: Lake Brommelille | Duan et al. 2021b |
| MW000967 | *Australapatemom* sp. | Cercaria | *Ampullaceana balthica* | Denmark: Lake Tjele Langsoe | Duan et al. 2021b |
| KY570947, KY570948 | *Australapatemom* sp. | Not specified | Not specified | USA, California, Big Pond - Palassou | Gordy et al. 2017 |
| MW000961 - MW000965  MW000945, MW000946, MW000950 - MW000960  MW000947 - MW000949 | *Australapatemom burti* | Cercaria | *Planorbis planorbis*  *Anisus vortex* | Denmark, Lake Tissoe  Denmark, Lake Furesoe  Denmark, Lake Esrum | Duan et al. 2021b |
| MN745949 | *Bolbophorus* sp. | Cercaria | *Bulinus ugandae* | Kenya, Lake Victoria | Outa et al. 2020 |
| MN080274 - MN080283, MN080288 | *Posthodiplostomum centrarchi* | Metacercaria | *Lepomis gibbosus* | Hungary | Cech et al. 2020 |
| MH521251 | *Posthodiplostomum centrarchi* | Adult | *Ardea herodias* | Canada, Hudson, Montreal area, QC | Locke et al. 2018 |
| MK604881 | *Posthodiplostomum* sp. | Metacercaria | *Tilapia sparrmanii* | South Africa, Boskop Dam | Hoogendoorn et al. 2019 |
| MH358392, MH358393 | *Posthodiplostomum nanum* | Metacercariae | *Poecilia reticulata* (experimental host) | Brazil, Minas Gerais | Lopez-Hernandez et al. 2018 |
| MG780487 | *Diplostomum pseudospathaceum* | Metacercaria | *Lota lota* | China | Duan 2017 (Direct submission) |
| MG780486 | *Diplostomum spathaceum* | Metacercaria | *Lota lota* | China | Duan 2017 (Direct submission) |
| KY951727 | *Ornithodiplostomum* sp. |  |  | Canada, St. Clair River, Point Edward | Blasco-Costa & Locke 2017 |
| KY462834 - KY462835, KY462838, | *Tylodelphys* sp. | Metacercaria | *Puntius sophore* | India | Chaudhary et al. 2017 |
| KC685368 | *Tylodelphys* sp. | Metacercaria | *Clarias gariepinus* | Tanzania, Lake Victoria | Chibwana et al. 2013 |
| FJ470401  FJ470398 | *Tylodelphys* sp. | Metacercaria | *Clarias gariepinus* | Tanzania, Ruvu River  Tanzania, Mwanza Gulf | Chibwana 2008 (Direct submission) |
| KY320573, KY320572 | *Crassiphialinae* sp. | Metacercaria | *Valencia letourneuxi* | Greece, Acheron Delta | Kalogianni et al. 2017 |
| KT728769, KT728773 | *Diplostomidae* sp. | Cercaria | *Biomphalaria obstructa* | USA, Noxubee County, MS | Rosser et al. 2016 |
| KJ137229 - KJ137231 | *Pharyngostomum cordatum* | Adult | *Felis catus* | China | Kang et al. 2014 (Direct submission) |
| **ITS Cyathocotylidae** |  |  |  |  |  |
| MT668947, MT668949, MT668951  MT668942, MT668943, MT668945, MT668946  MT668941, MT668944 | *‘Holostephanus* sp.*’* | Metacercaria | *Cyprinus carpio*  *Tinca tinca*  *Scardinius erythrophthalmus* | Hungary  Italy | Cech et al. 2020 |
| PP093043, PP093044, PP093045  PP093040  PP093041 | *Paracoenogonimus ovatus* | Adult  Metacercaria | *Circus aeruginosus*  *Rutilus rutilus*  *Pelecus cultratus* | Russia Astrakhan Region  Russia, Ladoga Lake | Sokolov et al. 2024 |
| PV482971 - PV482973 | *Paracoenogonimus ovatus* | Metacercaria | *Tinca tinca* | Germany | This study |
| OM755735 | *Holostephanus dubinini* | Adult | *Canis lupus familiaris* (experimental host) | Egypt | Mohamed et al. 2022 (Direct submission) |
| MT668940 | *Holostephanus dubinini* | Metacercaria | *Scardinius erythrophthalm*us | Italy | Cech et al. 2020 |
| MH521249 | *Cyathocotyle prussica* | Metacercaria | *Gasterosteus aculeatus* | Germany, Grosser Ploner See | Locke et al. 2018 |
| PP093046 | *Georduboisia* cf. *teganuma* | Adult | *Podiceps cristatus* | Russia, Astrakhan Region | Sokolov et al. 2024 |
| OP348882 | *Mesostephanus* sp. | Adult | *Columba livia* | Egypt, Ismailia | Abuzeid & Li 2022 (Direct submission) |
| KY851309 | *Neodiplostomum americanum* | Adult | *Megascops asio* | USA, Mississippi | Woodyard et al. 2017 |
| MZ616381 | *Diplostomum phoxini* | Metacercaria | *Phoxinus phoxinus* | Germany, River Ruhr | Schwelm et al. 2021 |

**References**

Achatz, T. J et al. Molecular phylogenetic analysis of *Neodiplostomum* and *Fibricola* (Digenea, Diplostomidae) does not support host-based systematics. *Parasitology*, **149** (4), 542–554. (2022).

Ai, L. et al. Sequences of internal transcribed spacers and two mitochondrial genes: effective genetic markers for *Metorchis orientalis*. J Anim Vet Ad, 9: 2371 – 2376. (2010). 10.3923/javaa.2010.2371.237

Besprozvannykh, V.V., Tatonova, Y.V. & Shumenko, P.G. Life cycle, morphology of developmental stages of *Metorchis ussuriensis* sp. nov. (Trematoda: Opisthorchiidae), and phylogenetic relationships with other opisthorchiids. *J. Zool. Syst. Evol. Res.*, **57**, 24–40 (2019)

Blasco-Costa, I., & Locke, S. A. Life History, Systematics and Evolution of the Diplostomoidea Poirier, 1886: Progress, Promises and Challenges Emerging From Molecular Studies. *Advances in parasitology*, **98**, 167–225. (2017). <https://doi.org/10.1016/bs.apar.2017.05.001>

Cai, X. Q. et al. Sequences and gene organization of the mitochondrial genomes of the liver flukes *Opisthorchis viverrini* and *Clonorchis sinensis* (Trematoda). *Parasitology research*, **110**(1), 235–243. (2012). https://doi.org/10.1007/s00436-011-2477-2

Cech, G. et al. Digenean trematodes in Hungarian freshwater aquacultures. *Food and waterborne parasitology* **22** (2020). e00101. <https://doi.org/10.1016/j.fawpar.2020.e00101>

Chaudhary, A., Gupta, S., Tripathi, R., & Singh, H. S. Morphological and molecular analyses of *Tylodelphys* spp. metacercaria (Trematoda: Diplostomidae) from the vitreous humour of two freshwater fish species, *Channa gachua* (Ham.) and *Puntius sophore* (Ham.). *Veterinary parasitology*, **244**, 64–70. (2017). https://doi.org/10.1016/j.vetpar.2017.07.016

Chibwana, F et al. A first insight into the barcodes for African diplostomids (Digenea: Diplostomidae): brain parasites in *Clarias gariepinus* (Siluriformes: Clariidae). *Infection, genetics and evolution: journal of molecular epidemiology and evolutionary genetics in infectious diseases*, **17**, 62–70. (2013). https://doi.org/10.1016/j.meegid.2013.03.037

Duan, Y et al. Eye fluke effects on Danish freshwater fish: Field and experimental investigations. *Journal of fish diseases*, **44**(11), 1785–1798. (2021a). https://doi.org/10.1111/jfd.13496

Duan, Y., Al-Jubury, A., Kania, P. W., & Buchmann, K. Trematode diversity reflecting the community structure of Danish freshwater systems: molecular clues. *Parasites & vectors*, **14**(1), 43. (2021b). <https://doi.org/10.1186/s13071-020-04536-x>

Gordy, M. A., Locke, S. A., Rawlings, T. A., Lapierre, A. R., & Hanington, P. C. Molecular and morphological evidence for nine species in North American *Australapatemon* (Sudarikov, 1959): a phylogeny expansion with description of the zygocercous *Australapatemon mclaughlini* n. sp. *Parasitology research* **116** (8), 2181–2198. (2017). https://doi.org/10.1007/s00436-017-5523-x

Hoogendoorn, C., Smit, N. J., & Kudlai, O. Molecular and morphological characterisation of four diplostomid metacercariae infecting *Tilapia sparrmanii* (Perciformes: Cichlidae) in the North West Province, South Africa. *Parasitology research*, **118**(5), 1403–1416. (2019). <https://doi.org/10.1007/s00436-019-06285-y>

Kang, S. et al. Molecular identification and phylogenetic analysis of nuclear rDNA sequences among three opisthorchid liver fluke species (Opisthorchiidae: Trematoda). *Parasitology international*, **57** (2), 191–197. (2008). https://doi.org/10.1016/j.parint.2007.12.007

Kalogianni, E. et al. Occurrence and effect of trematode metacercariae in two endangered killifishes from Greece. *Parasitology research*, **116** (11), 3007–3018. (2017). https://doi.org/10.1007/s00436-017-5610-z

Locke, S. A., Daniel McLaughlin, J., & Marcogliese, D. J. DNA barcodes show cryptic diversity and a potential physiological basis for host specificity among Diplostomoidea (Platyhelminthes: Digenea) parasitizing freshwater fishes in the St. Lawrence River, Canada. *Molecular ecology,* **19**(13), 2813–2827. (2010). <https://doi.org/10.1111/j.1365-294X.2010.04713.x>

Locke, S. A., McLaughlin, J. D., Lapierre, A. R., Johnson, P. T., & Marcogliese, D. J. Linking larvae and adults of *Apharyngostrigea cornu, Hysteromorpha triloba*, and *Alaria mustelae* (Diplostomoidea: Digenea) using molecular data. *The Journal of parasitology*, **97** (5), 846–851. (2011). https://doi.org/10.1645/GE-2775.1

Locke, S. et al. Validity of the Diplostomoidea and Diplostomida (Digenea, Platyhelminthes) upheld in phylogenomic analysis. *Int. J. Parasitol.,* **48**, 1043–1059 (2018).

López-Hernández, D., Locke, S. A., Melo, A. L., Rabelo, É. M. L., & Pinto, H. A. Molecular, morphological and experimental assessment of the life cycle of *Posthodiplostomum nanum* Dubois, 1937 (Trematoda: Diplostomidae) from Brazil, with phylogenetic evidence of the paraphyly of the genus *Posthodiplostomum* Dubois, 1936. *Infection, genetics and evolution: journal of molecular epidemiology and evolutionary genetics in infectious diseases*, **63**, 95–103. (2018). https://doi.org/10.1016/j.meegid.2018.05.010

López-Hernández, D. et al. Molecular, morphological and experimental-infection studies of cercariae of five species in the superfamily Diplostomoidea (Trematoda: Digenea) infecting *Biomphalaria straminea* (Mollusca: Planorbidae) in Brazil. Acta trop., **199**, 105082 (2019). https://doi.org/10.1016/j.actatropica.2019.105082

Ma, Jet al. Characterization of the mitochondrial genome sequences of the liver fluke *Amphimerus* sp. (Trematoda: Opisthorchiidae) from Ecuador and phylogenetic implications. *Acta tropica*, **195**, 90–96. (2019). https://doi.org/10.1016/j.actatropica.2019.04.025

Outa, J. O., Sattmann, H., Köhsler, M., Walochnik, J., & Jirsa, F. Diversity of digenean trematode larvae in snails from Lake Victoria, Kenya: First reports and bioindicative aspects. *Acta tropica*, **206**, 105437. (2020). https://doi.org/10.1016/j.actatropica.2020.105437

Rosser, T. et al. Characterization of the Life Cycle of a Fish Eye Fluke, *Austrodiplostomum ostrowskiae* (Digenea: Diplostomidae), with Notes on Two Other Diplostomids Infecting *Biomphalaria havanensis* (Mollusca: Planorbidae) from Catfish Aquaculture Ponds in Mississippi, USA. *The Journal of parasitology*, **102**(2), 260–274. (2016). https://doi.org/10.1645/15-850

Schwelm, J., Georgieva, S., Grabner, D., Kostadinova, A., & Sures, B. Molecular and morphological characterisation of *Diplostomum phoxini* (Faust, 1918) with a revised classification and an updated nomenclature of the species-level lineages of*Diplostomum* (Digenea: Diplostomidae) sequenced worldwide. *Parasitology,* **148**(13). (2021). 1648–1664. <https://doi.org/10.1017/S0031182021001372>

Sereno-Uribe, A., López-Jimenez, A., Andrade-Gómez, L. & García-Varela, M. A morphological and molecular study of adults and metacercariae *of Hysteromorpha triloba* (Rudolpi, 1819), Lutz 1931 (Diplostomidae) from the Neotropical region. *J. Helminthol.*, **93**, 91–99 (2019).

Shekhovtsov, S. V., Katokhin, A. V., Kolchanov, N. A., & Mordvinov, V. A. The complete mitochondrial genomes of the liver flukes *Opisthorchis felineus* and *Clonorchis sinensis* (Trematoda). *Parasitology international*, **59**(1), 100–103. (2010). <https://doi.org/10.1016/j.parint.2009.10.012>

Sherrard-Smith, E. et al. Distribution and molecular phylogeny of biliary trematodes (Opisthorchiidae) infecting native *Lutra lutra* and alien *Neovison vison* across Europe. *Parasitol. Int*., **65**, 163-170 (2016).

Sokolov, S., Kalmykov, A., Frolov, E. & Atopkin, D. Taxonomic myths and phylogenetic realities in the systematics of the Opisthorchiidae (Trematoda). *Zoologica Scripta,* **51**, 232–245 (2022).

Sokolov, S.G., Vlasenkov, S.A., Bugmyrin, S.V., Kalmykov, A.P. & Lebedeva, D.I. Phylogeny and morphology of some European cyathocotylid digeneans (Trematoda: Diplostomoidea). *Journal of Helminthology*, **98**, e44, 1–11 (2024) https://doi.org/10.1017/S0022149X24000348.

Suleman, Ma, J., Khan, M. S., Sun, M. M., Muhammad, N., He, J. J., & Zhu, X. Q. Mitochondrial and nuclear ribosomal DNA dataset suggests that *Hepatiarius sudarikovi* Feizullaev, 1961 is a member of the genus *Opisthorchis* Blanchard, 1895 (Digenea: Opisthorchiidae). *Parasitology research*, **118**(3), 807–815. (2019). https://doi.org/10.1007/s00436-019-06227-8

Tatonova, Y. V., Besprozvannykh, V. V., Katugina, L. O., Solodovnik, D. A., & Nguyen, H. M. Morphological and molecular data for highly pathogenic avian parasite *Erschoviorchis anuiensis* sp. n. and phylogenetic relationships within the Opisthorchiidae (Trematoda). *Parasitology international*, **75**, 102055. (2020). <https://doi.org/10.1016/j.parint.2020.102055>

Wang, Y., Li, X., Sun, Q., Gong, P., Zhang, N., Zhang, X., Wang, X., Li, G., & Li, J. First case report of *Metorchis orientalis* from Black Swan. *International Journal for Parasitology: Parasites and Wildlife*, **13**, 7–12. (2020). https://doi.org/10.1016/j.ijppaw.2020.07.011

Woodyard, E. T., Rosser, T. G., & Griffin, M. J. New data on *Neodiplostomum americanum* Chandler and Rausch, 1947 (Digenea: Diplostomidae), in the Great Horned Owl *Bubo virginianus* Gmelin, 1788 and the Eastern Screech Owl *Megascops asio* Linnaeus, 1758 in Mississippi, USA. *Parasitology research*,**116**(8). (2017). 2075–2089. https://doi.org/10.1007/s00436-017-5503-1
